# AGE-STRATIFIED INVESTIGATION OF SLEEP DISORDERS AND PTSD PREVALENCE AMONG VIETNAM VETERANS

**DOI:** 10.1093/geroni/igad104.3170

**Published:** 2023-12-21

**Authors:** Thomas Chacko, Sara Mithani, Melissa Bunker, Charity Breneman, Gordon Broderick

**Affiliations:** Rochester Regional Health, Rochester, New York, United States; UT Health San Antonio, San Antonio, Texas, United States; The University of Texas at San Antonio, San Antonio, Texas, United States; War Related Illness and Injury Study Center, VA, Washington, District of Columbia, United States; Rochester Regional Health, Rochester, New York, United States

## Abstract

Military Veterans often experience a range of sleep disorders that significantly impact their physical and psychological wellbeing. Current study describes prevalence and age differences (≥65 to ≤74yrs; n =193)) vs. (≥75 to ≤ 90yrs; n =157) across insomnia, obstructive sleep apnea (OSA), nightmare disorder (ND), and posttraumatic stress disorder (PTSD). Vietnam Veterans (N = 350; AgeMean= 74.4 [SD= 3.6]) completed a survey (online or paper) of their sleep experiences and PTSD as part of a cross-sectional study. Veterans were mostly White (95%), male (97.7%), from the Army (55%), with some or two-year college (43%), and currently married (76%). Insomnia was screened positive in 27.7% of the Veterans, OSA in 58.6%, ND in 5.7%, and PTSD in 18.6%. There were significant mean differences in insomnia and PTSD across age groups (t(348) = 2.43, p = .016) and (t(348) = 2.06, p =.04), respectively, with younger age group showing higher insomnia, and PTSD. There were no significant mean differences in OSA and ND. Pearson correlation analyses yielded important associations. Insomnia was significantly positively correlated with PTSD (r= .60), OSA (r= .33), and ND (r= .51). PTSD was significantly positively correlated with OSA (r= .30) and ND (r= .67). ND was significantly positively correlated with OSA (r= .22). There were no significant differences in the associations across the two age groups. Recognizing and addressing various sleep disorders and comorbid PTSD are crucial to improving the quality of life of Veterans. Results emphasize need for comprehensive assessment of sleep disorders and PTSD among Veterans.

